# Psychoeducation of Higher Education Students With Using of The Resource-Oriented Model of Resiliency in Ukrainian War Conditions

**DOI:** 10.1192/j.eurpsy.2026.11698

**Published:** 2026-07-31

**Authors:** V. Ogorenko, T. Shusterman, A. Nikolenko, V. Kokashynskyi

**Affiliations:** Psychiatry, Narcology and Medical Psychology, DSMU, Dnipro, Ukraine

## Abstract

**Introduction:**

Higher education students are a vulnerable category of the population who needs psychological support and accompaniment. The aim of the study was to identify the modalities of resiliency of higher education students in Ukrainian war conditions with the subsequent development of psychoeducational measures regarding their more effective and balanced use.

**Objectives:**

Total number of participants is 174 higher education students: 144 students from the Dnipro State Medical University (main group) and 30 – Ukrainian State University of Science and Technology (comparison group).

**Methods:**

The psychodiagnostic method of the resource-oriented model of resiliency «BASIC Ph» was used.

**Results:**

The predominance of «Cognition» in the participants of both groups with insufficient use of the modality «Social support» (main group) and «Affect» (comparison group) has been identified. Psychoeducation for higher education students included lectures and individual conversations upon request. The «BASIC Ph» model of stress resiliency was utilized as a framework for determining optimal human resource utilization during crises, with six modalities forming individual coping styles. It was crucial to identify dominant modalities and develop those that were less effective.

**Conclusions:**

The identified stress resiliency modalities provided a conceptual basis for developing of psychoeducational measures for higher education students. During psychoeducation resiliency models have been analyzed, interpreted, and balanced. The study’s results should be taken into account at providing of psychological support efforts for higher education students in war and post-war periods in Ukraine.

**Disclosure of Interest:**

None Declared

